# PlantPathMarks (PPMdb): an interactive hub for pathways-based markers in plant genomes

**DOI:** 10.1038/s41598-021-00504-2

**Published:** 2021-10-29

**Authors:** Morad M. Mokhtar, Achraf El Allali, Mohamed-Elamir F. Hegazy, Mohamed A. M. Atia

**Affiliations:** 1 African Genome Center, Mohammed VI Polytechnic University, Ben Guerir, Morocco; 2 Chemistry of Medicinal Plants Department, National Research Centre, Giza, 12622 Egypt; 3 Molecular Genetics and Genome Mapping Laboratory, Genome Mapping Department, Agricultural Genetic Engineering Research Institute (AGERI), Agriculture Research Center (ARC), Giza, 12619 Egypt

**Keywords:** Computational biology and bioinformatics, Data mining, Databases, Functional clustering

## Abstract

Over the past decade, the problem of finding an efficient gene-targeting marker set or signature for plant trait characterization has remained challenging. Many databases focusing on pathway mining have been released with one major deficiency, as they lack to develop marker sets that target only genes controlling a specific pathway or certain biological process. Herein, we present the PlantPathMarks database (PPMdb) as a comprehensive, web-based, user-friendly, and interactive hub for pathway-based markers in plant genomes. Based on our newly developed pathway gene set mining approach, two novel pathway-based marker systems called pathway gene-targeted markers (PGTMs) and pathway microsatellite-targeted markers (PMTMs) were developed as a novel class of annotation-based markers. In the PPMdb database, 2,690,742 pathway-based markers reflecting 9,894 marker panels were developed across 82 plant genomes. The markers include 691,555 PGTMs and 1,999,187 PMTMs. Across these genomes, 165,378 enzyme-coding genes were mapped against 126 KEGG reference pathway maps. PPMdb is furnished with three interactive visualization tools (Map Browse, JBrowse and Species Comparison) to visualize, map, and compare the developed markers over their KEGG reference pathway maps. All the stored marker panels can be freely downloaded. PPMdb promises to create a radical shift in the paradigm of the area of molecular marker research. The use of PPMdb as a mega-tool represents an impediment for non-bioinformatician plant scientists and breeders. PPMdb is freely available at http://ppmdb.easyomics.org.

## Introduction

Today, an enormous amount of released biodata collections stimulates the development of computerized applications worldwide^1^. Inspiration drives scientists to develop intelligent approaches to extract hidden knowledge behind these massive amounts of data. Biodata mining attempts to find novel, reliable, useful, and meaningful insights from these vast amounts of data. Over the last decades, the scope of biodata mining has expanded from genome-mining to phenome-mining approaches^2^. Its applications outside of basic research have become extensive to study plants’ genetic diversity and improve economic crop breeding programs. Generally, plants produce various chemical compounds involving nutritional or medicinal benefits^3^. Our understanding of plant natural products’ biological pathways is still insufficient; however, scientists assume that genomic and metabolomic information can provide clues about unidentified enzymes and reactions involved in a particular biosynthesis process^4^.

Plants have undergone complicated evolutionary events that have resulted naturally or are due to human-made plant breeding experiments and finally lead to polyploidy or genome duplication^3^. This duplication drives the availability of genes either as homologous or paralogous. Due to the phylogenetic relationship of homologous genes, they continue to preserve their core functions. Gene products are often mapped to many pathways revealing the same catalyst properties under different reaction conditions or showing the same reaction but in distinct subcellular locations^5^. Such an association provides a mechanism to study genes’ roles; their expression may be regulated spatially or temporally due to evolutionary implications on plants, gene products, gene functions, and, finally, developmental stages^1^.

A biological pathway is broadly defined as a series of actions among cell molecules that lead to a particular product or cellular change. It can also trigger novel molecule assembly, turn genes on and off, or drive a cell to move. Any biological pathway comprises a cluster of genes that exhibit identical or complementary biological functions^6^. Nowadays, pathway analysis is a flourishing research area in systems biology. It relies on extracting knowledge from raw data generated from high-throughput sequencing technologies by building a model that describes and summarizes underlying biological processes. These high-throughput technologies mostly provide a list of differentially expressed genes (DEGs) between a control and a case of interest. Unfortunately, generated DEG data are usually removed from their biological context, causing the resultant genes to require further validation against biological pathways to prove their biological significance^7^. Notably, each of these genes may be a part of many pathways, which usually start with the expression of a gene of a particular ligand and is terminated with an alteration in the concentration of a specific signaling micro-molecule^8^.

The dilemma of plant trait/metabolite characterization and improvement based on single or few genes targeting marker selections remains challenging, and many approaches have been developed to tackle these issues^9^. For decades, massive studies have aimed to determine a few differentially expressed marker genes participating in certain natural products’ biosynthesis processes or conferring a particular phenotype^10–12^. Nevertheless, these methods may not be accurate and may lack in capturing an in-depth snapshot of biological processes. Therefore, studies focusing on gene sets belonging to a particular pathway to investigate and explain phenotypic changes have gained increased popularity. These methods are more acceptable, as they assess the significance of a group of predefined genes with shared biological functions to explain specific phenotypes^13–15^.

In contrast to traditional gene-based methods, pathway-based methods combined with biological knowledge can help gain a better understanding of functional insights into phenotypic differences. Pathway-mining approaches attempt to rectify such drawbacks by utilizing the available biological knowledge base about the structures and operations of biological pathways accompanying computational methods^5^. Therefore, instead of focusing on specific gene-based marker development, our proposed pathway gene set mining (PGM) approach is critically more appropriate and promises to create a radical shift in the paradigm of molecular markers as a research area. The dramatically increasing number of sequenced plant genomes is supposed to reveal more basic architectural principles of biosynthetic pathways for generating chemical diversity of natural products. Based on released knowledge on interactions between genes and their phenotypic reflections, many publicly available databases have been developed for storing this pathway mining information^16^.

After numerous plant genomes are fully sequenced, scientists often search for an efficient scheme that can analyze their data and answer certain biological questions^3^. Common types of analyses include pathway enrichment analysis and the analysis of gene ontology and gene–gene interactions. Over the last decade, many powerful resources for studying metabolic pathways in plants, such as KEGG^17^, Gramene^18^, Plant Reactome^19^, MapMan^20^, MetaCyc^21^, Plant Metabolic Network^22^, and BioCyc^23^, have been developed. These databases try their best to integrate the most available information when they were initially launched. Their construction process includes manual curation and computational efforts, regular screening of newly released publications, and updating of interactors. Despite extraordinary efforts, no single database is fully equipped with all the preferred information and may not accommodate all the necessary bioinformatics tools. Notably, all these resources have the significant limitation of missing genome-scale marker sets that target only the genes responsible for a specific pathway or that are involved in/control a particular biological process at the level.

Gene sets involved in a particular pathway often work together in an orchestral pattern to reflect a specific biological function. Herein, we proposed a novel PGM pipeline/approach to develop a specific marker set for each pathway at the genome-scale level in plant genomes.

Microsatellites, also known as simple sequence repeats (SSRs), are a set of one to six nucleotides that are repeated tandemly and classified either into perfect, imperfect, or composite repeats^24,25^. Due to their unique characteristics (hypervariability, multi-allelic nature, high reproducibility, Mendelian inheritance, and overall high abundance in plant genomes), as well as their essential roles in many biological functions (modulating transcription factor binding, tuning knobs of gene expression levels, organizing chromatin, nucleosome positioning, acting as recombination hot spots, and facilitating unusual structural conformations), microsatellites have become one of the most preferred choices among all genetic markers because they afford a molecular basis for plant genome evolution and demonstrate fast acclimatization to many abiotic stresses^26^. Therefore, we proposed a mining schema of microsatellites within gene sets involved in all plant genome pathways as a robust and functional approach to develop a novel marker system called pathway microsatellite-targeted markers (PMTMs).

Ultimately, this study aims to develop a comprehensive hub for two novel pathway-based marker systems coined pathway gene-targeted markers (PGTMs) and pathway microsatellite-targeted markers (PMTMs) in plant genomes. Furthermore, the developed markers were mapped against reference KEGG pathway maps interactively. The use of these powerful portal represents an impediment for non-bioinformatician plant scientists and breeders.

## Materials and methods

### Data collection

To build a comprehensive pathway-based marker database in plants, we retrieved complete genome sequences and annotations of 82 various plant species, including 72 eudicots and 10 monocots. The sequences and annotations were downloaded as GenBank files from NCBI’s FTP site (ftp://ftp.ncbi.nlm.nih.gov/). Furthermore, we downloaded all KEGG gene annotations available on the KEGG database of these plant genomes (approximately 2,296,000 genes) in addition to all KEGG pathway reference maps (https://www.genome.jp/kegg/).

### Data analysis and database construction

**Figure 1:**
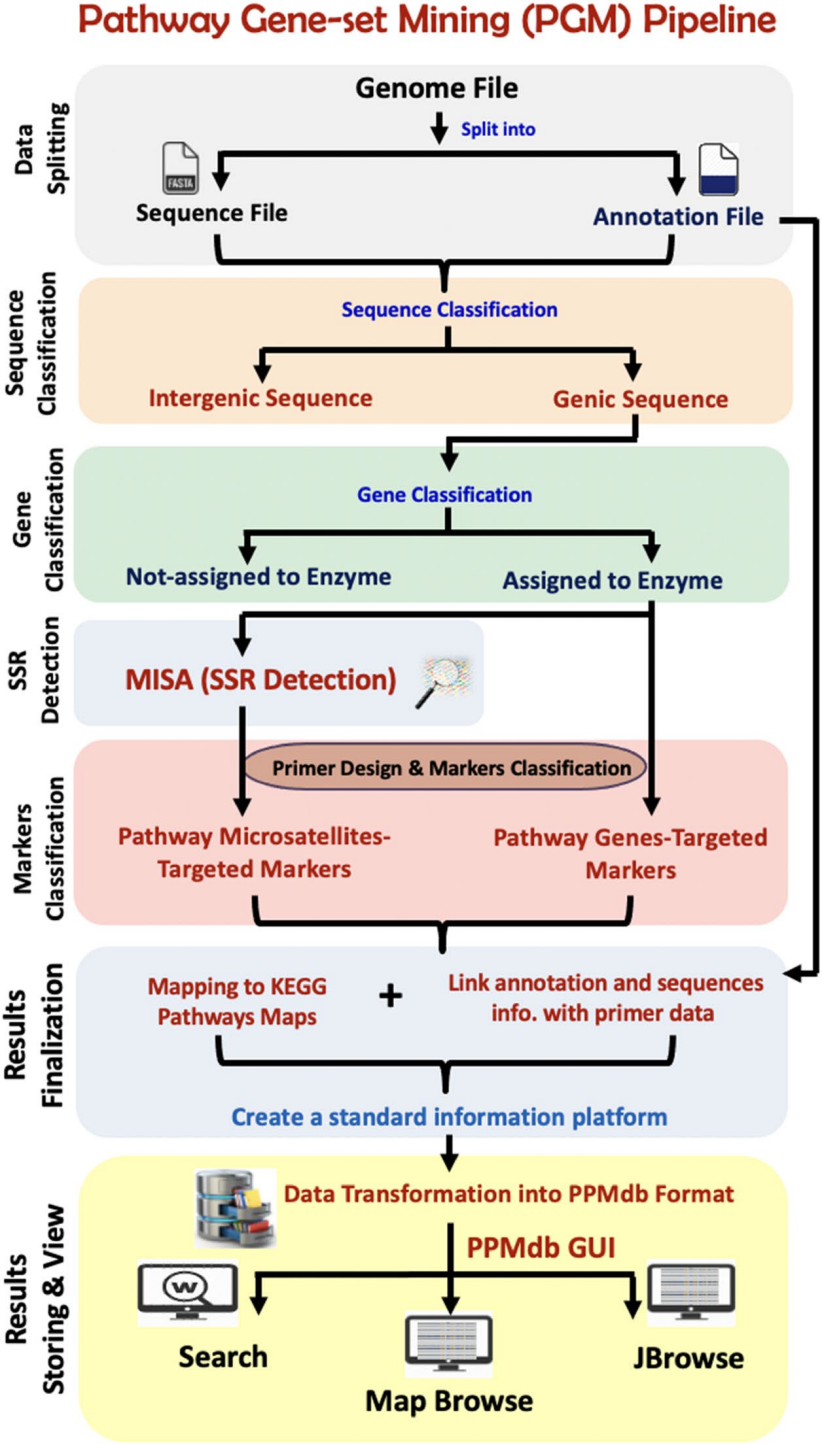
The workflow of pathway gene-set mining (PGM) pipeline.

To construct the PlantPathMarks (PPMdb) core database, we integrated two powerful tools called MIcroSAtellite identification (MISA)^27^ and primer3^28^ into our in-house-developed “Perl and Shell” scripts to strap all standalone bioinformatics analysis steps in one main pipeline called the PGM pipeline. This pipeline was built to develop two novel marker systems called PGTM and PMTM. This pipeline was implemented to construct our PPMdb sub-databases through seven main steps: (a) splitting the retrieved genomic data into sequence and annotation files, (b) classifying genomic sequence into genic or intergenic, (c) sub-classifying genes into enzyme coding or non-enzyme coding, (d) mining microsatellites on enzyme-coding genes only (this step exclusive to PMTM), (e) designing primers and classifying markers into PMTM or PGTM, (f) mapping all designed primers against KEGG reference maps^17^ besides linking associated information (sequence and annotation) to all pathway-mapped markers, and (g) data transforming into the PPMdb database format and implementing all datasets into the PPMdb web interface (Fig. 1).

For effective marker development, we used a straightforward strategy to boost data analysis and marker generation processes. Before we began, we established in-house “Perl and Shell” scripts to convert all retrieved genome sequence and annotation data and unify input and output files used for the development of the PPMdb database.

Initially, the data retrieved from the 82 plant genomes were dissected to classify each genome into genic and intergenic according to the genome’s annotation information. This classification step was achieved with the aid of the gene-finding format (GFF) file for each genome. The gene ID list for each plant was further searched against the KEGG database (https://www.genome.jp/kegg/ genes.html) to sub-classify genes into enzyme coding or non-enzyme coding. Thereafter, perfect and compound microsatellite motifs were identified using the MISA tool^27^ according to the following parameters: mono- (≥ 9), di- (≥ 4), tri- (≥ 3), tetra- (≥ 3), penta- (≥ 2), and hexa-nucleotide (≥ 2). The parameter was set as ≥ 2 repeats interrupted by ≤ 20 bp for the characterization of compound microsatellite motifs^29^.

PMTM and PGTM primers were then designed for all enzyme-coding genes for all plant genomes using the Primer3 software^28^ according to the following criteria: a) optimal primer length of 20 bp; b) optimal melting temperature of 55 °C; c) product size range of 90–500 bp for PMTM primers and 100–1000 bp for PGTM primers; and d) a 50% G/C content. Then, e) a unique primer ID was assigned.

All developed PMTM and PGTM primers were mapped against the KEGG pathway reference maps (https://www.genome.jp/kegg/pathway.html) by using in-house “Perl and Shell” scripts to integrate and localize our developed PMTM and PGTM primers and build a map set of marker-enriched pathways for each plant. Each map contains a pathway ID, pathway image, IDs of mapped enzymes (highlighted), PMTM/PGTM markers associated with mapped enzymes, annotation information of each marker, and other information related to this primer (Tm, GC%, length, etc.). All developed maps were visualized in an attractive user form using Jquery ImageMapster Plugin^30^.

The JBrowse tool^31^ was also integrated into our PPMdb database as a powerful web application for genome analysis and visualization. Herein, it was used to map and browse the identified microsatellite motifs and developed PMTMs and PGTMs and their linked information for each organism.

All generated data of PMTMs, PGTMs, and maps were further processed to build a standard information platform for all marker types before integrating them into the PPMdb SQL database. With the aid of the in-house “Perl and Shell” scripts, we batch processed all developed markers, maps, and any associated data and converted them into a consistent format. Additionally, we uploaded these scripts to online open-source housing website (GitHub) to make it available for all users at the following link (https://github.com/MoradMMokhtar/PlantPathMarks-Scripts.git). The PPMdb database was developed with aid of the LPPM (Linux + Perl + PHP + MySQL) web application platform; finally, JavaScript, CSS, and HTML languages were used to design a user-friendly interactive web interface.

## Features and utilities

### Database interface

PPMdb presents an interactive, user-friendly portal well equipped with many features to enable users to search and download PMTMs and PGTMs across 82 plant genomes. PPMdb provides users with a navigation bar designed to help access the PPMdb database sections and tools in a responsive and convenient way. The PPMdb data can be straightforwardly browsed and retrieved via nine interactive pages: Home (PPMdb Quick-Access), Database Search, Map Browse, JBrowse, Statistics, Data Resources, Bulk Download, Species Comparisons and Manual. Under these pages, plants are taxonomically grouped or sorted alphabetically to improve the convenience of exploration and selection.

**Figure 2:**
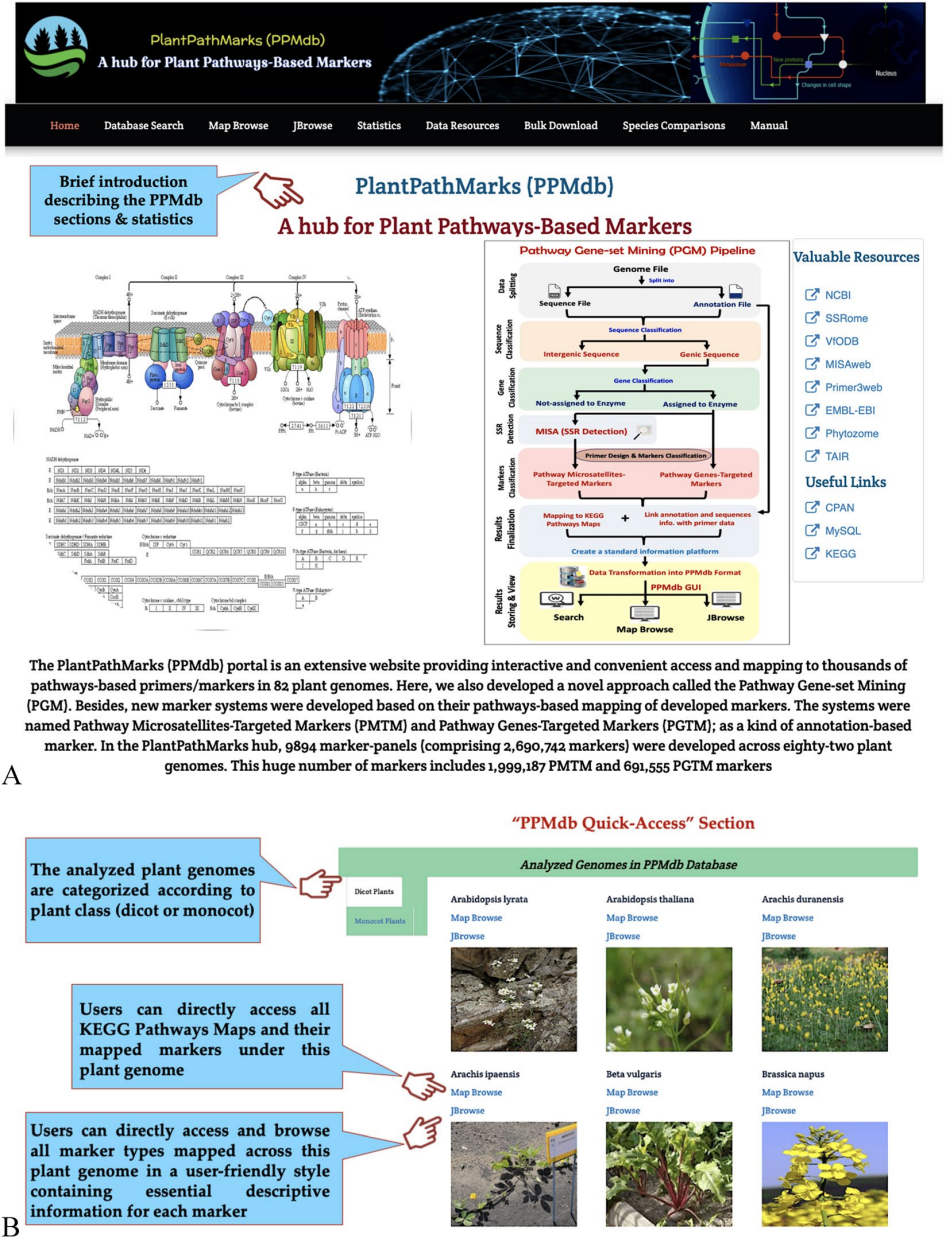
Screenshot of the PPMdb homepage (**A**) Database description and (**B**) PPMdb Quick-Access section.

The Home page introduces PPMdb as a comprehensive database along with its PGM pipeline by describing the PPMdb database sections, in addition to providing users with a “PPMdb Quick-Access; Analyzed Genomes in PPMdb Database Section” option for all analyzed plant genomes. In the “PPMdb Quick-Access” section, plant genomes analyzed in the PPMdb database are categorized according to plant type/class (dicot plants or monocot plants). Under each group, interactively, the plants are sorted alphabetically to enable straightforward access and searches within each plant genome independently through “Map Browse” or “JBrowse” quick-access links (Fig. 2).

**Figure 3:**
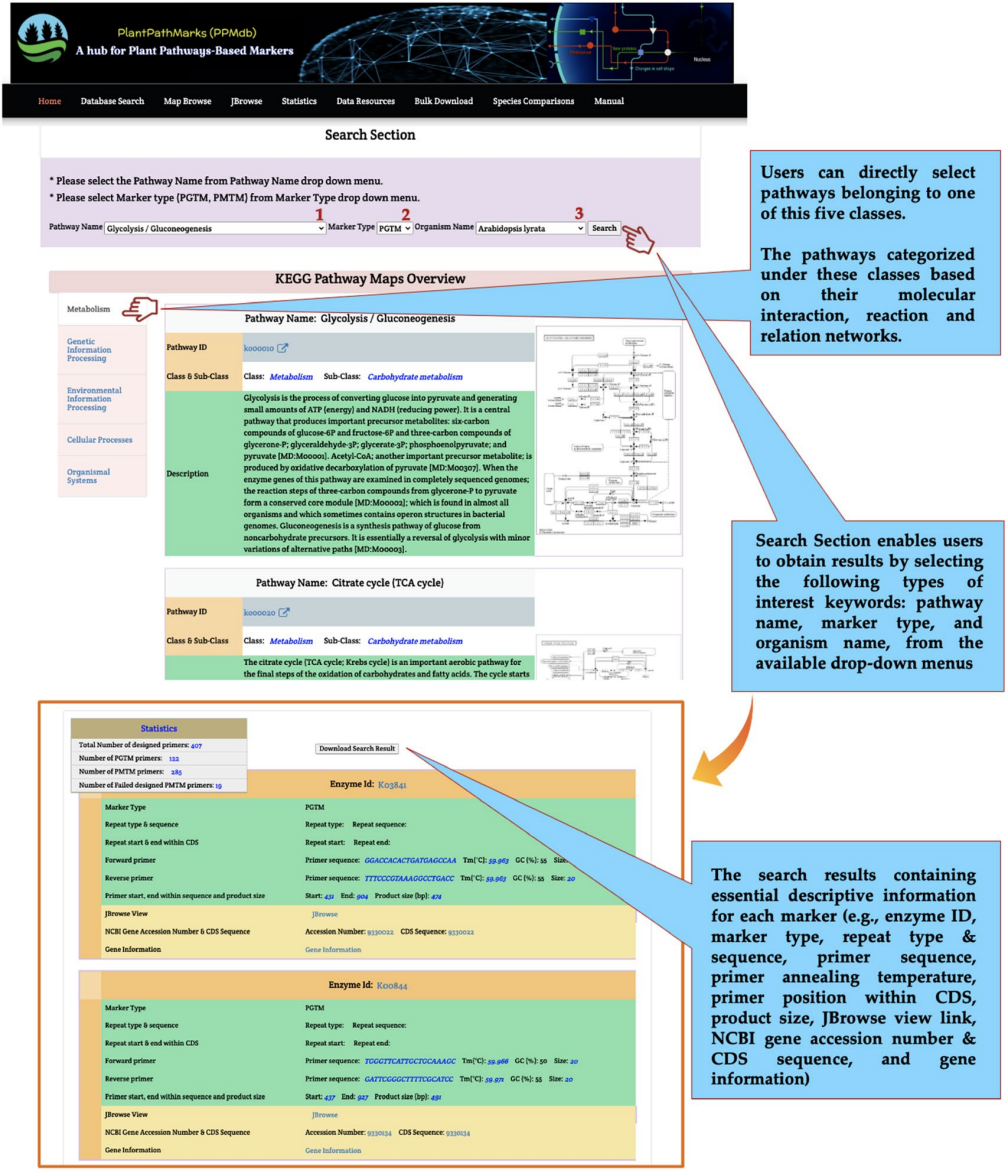
Screenshot of the PPMdb “Database Search” page layout.

The Database Search page provides users with a searching utility within two main sections: (a) the Search section, which enables users to obtain results by selecting the following types of interest keywords: pathway name (e.g., Glycolysis), marker type (e.g., PGTM), and organism name (e.g., *Arabidopsis lyrata*), from the available drop-down menus to effortlessly access and retrieve all marker and map data stored in the PPMdb database. (b) The KEGG Pathway Maps Overview section, which provides users the necessary information for each pathway, such as pathway ID (hyperlinked to KEGG ref. pathways), pathway class (*e.g*., metabolism), pathway sub-class (e.g., carbohydrate metabolism), and pathway map. The search results of this section are designed in a user-friendly style containing essential descriptive information for each marker (e.g., enzyme ID, marker type, repeat type and sequence [exclusive to PMTM], primer sequence, primer annealing temperature, primer position within CDS, product size, JBrowse view link, NCBI gene accession number and CDS sequence, and gene information) (Fig. 3).

In addition, general statistic tables are presented to summarize the total number of designed primers, number of PGTM primers, number of PMTM primers, and number of failed designed PMTM primers within the selected pathway of a particular plant. All search results can be downloaded freely for each plant straightforwardly as a CSV file (Fig. 3).

**Figure 4:**
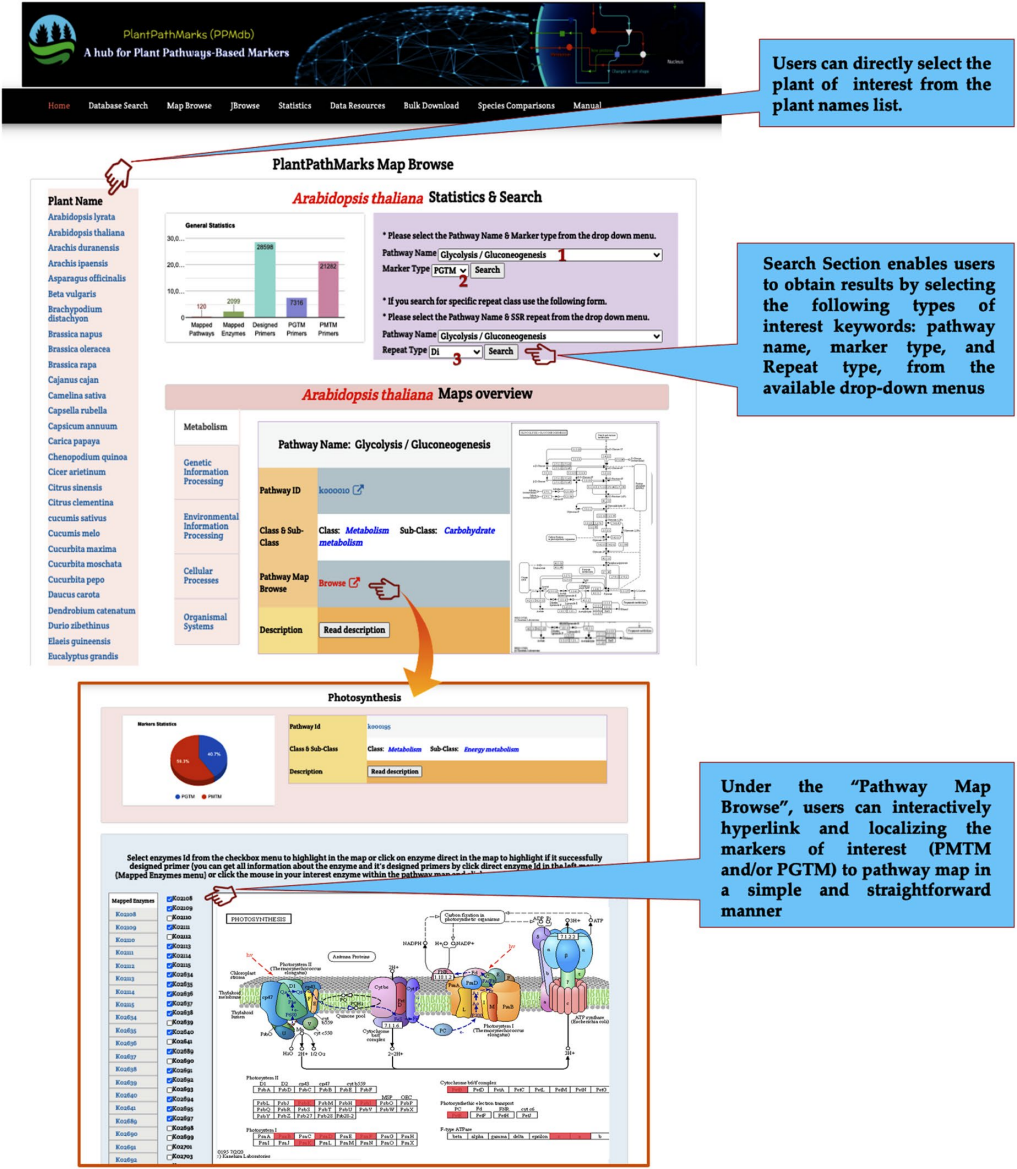
Screenshot of the PPMdb database “Map Browse” section including page layout and Pathway markers mapping layout.

The Map Browse page offers users with searching utility in two styles inside the same page: (a) selecting the plant of interest from the page-side plant list for a direct access of pathways and markers, (b) selecting the plant of interest from drop-down menus to directly access and obtain all markers and maps. In both styles, the search results are presented in a convenient, visualization-supported, and well-dissected manner involving essential information for each pathway, such as pathway ID (hyperlinked to KEGG ref. pathways), pathway class (e.g., metabolism), pathway sub-class (e.g., carbohydrate metabolism), pathway map browse (hyperlinked to interactive pathway map localizing all developed PMTM and PGTM markers on the pathway map), pathway description, and pathway map (Fig. 4).

**Figure 5:**
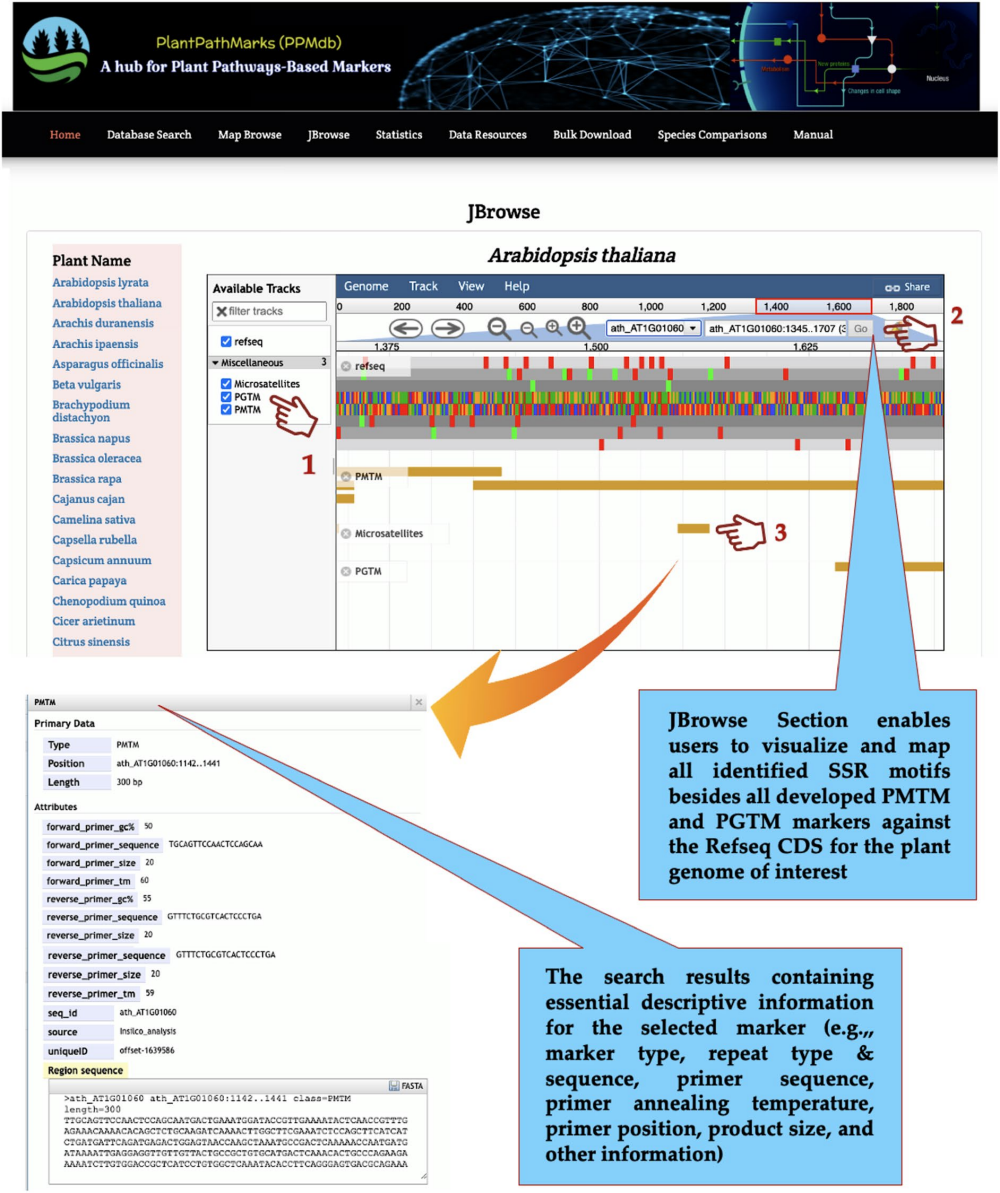
Screenshot of the PPMdb database “JBrowse” page.

The “JBrowse” page enables the users to visualize and map all identified microsatellite motifs besides all developed PMTM and PGTM markers against the Refseq CDS for each plant genome by selecting the plant of interest from the page-side plant list. The mapped microsatellite motifs and developed PMTM/PGTM markers were linked to its essential information (Fig. 5).

The ‘Statistics’ page was designed to provide users a primary indication of the taxonomy ID, number of mapped pathways, number of mapped enzymes, number of designed primers, number of PGTM primers, number of PMTM primers, and number of failed designed PMTM primers for each plant species stored in the PPMdb database.

The Data Resources page provides users with hyperlinks of all types/formats of the data utilized to build the PPMdb database. The page includes hyperlinks of taxonomy ID, Genbank file, Fasta file, GFF file, and KEGG gene annotation for each plant species analyzed within the PPMdb.

The Bulk Download page enables users to download all developed primers and their secondary information under a specific pathway in the organism of interest through the selection of organism name (e.g., *Arabidopsis lyrata*), pathway name (e.g., Glycolysis/Gluconeogenesis), and marker type (e.g., PGTM), from the drop-down menus straightforwardly in a convenient manner.

The Species Comparisons page offers users a powerful utility to compare two or three organisms in a particular pathway. The comparison results are provided under three main sections: general statistics, marker statistics, and distribution of the various SSR classes. The general statistics section layouts the numbers of mapped pathways, mapped enzymes, total designed primers, number of designed PGTM and PMTM primers under a particular pathway between the organisms of interest. Furthermore, the page offers a simple comparison of the distribution of various SSR classes within the pathway of interest. All comparisons are offered in a simple and effective visualization style.

## Statistics and discussion

### PPMdb database statistics

**Figure 6:**
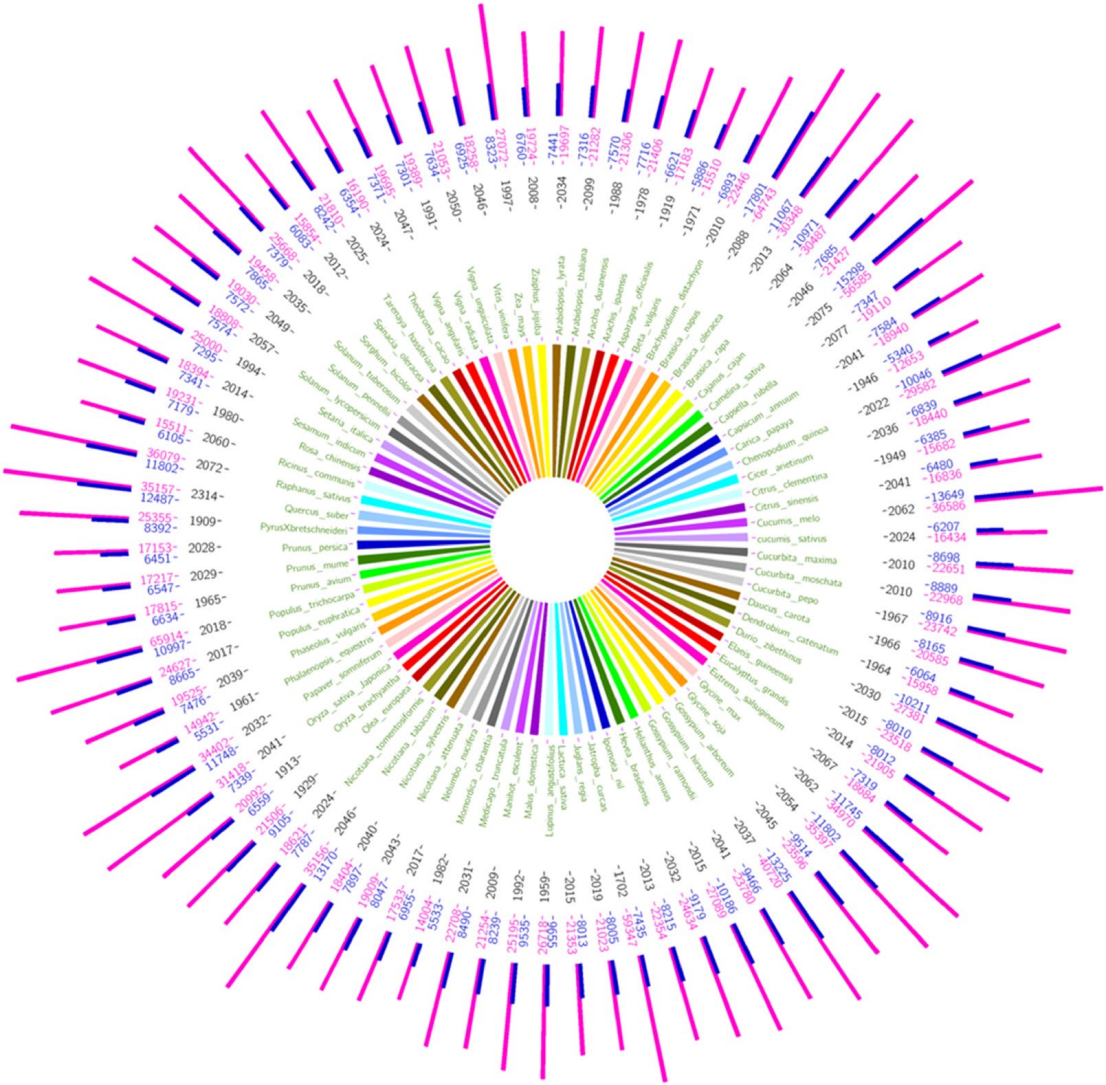
Statistical layout of the 82 analyzed plant genomes. The middle part shows the plants’ scientific names (each plant is assigned to a unique color). The black-colored numbers refer to the number of mapped enzymes within each plant genome, where the blue-colored numbers refer to the number of developed PGTMs, and the purple-colored numbers refer to the number of developed PMTMs.

As of January 2021, the PPMdb was launched and consisted of 2.7 million pathway-based markers distributed over 9894 marker panels developed across 82 plant genomes. Across these genomes, 165,378 enzyme-coding genes were mapped against 126 KEGG reference pathway maps. The SSR mining of all enzyme-coding genes identified 3,471,782 SSR motifs, including 2,844,501 perfect motifs and 627,281 compound motifs across the 82 plant genomes. Moreover, a total of 691,555 PGTMs and 1,999,187 PMTMs were developed (Fig. 6). This massive number of developed pathway-based markers have been mapped and supplemented with all essential information to offer users a modern version of markers called “pathway gene set markers”. All developed molecular markers for the 82 plant species are saved in separate backend tables for each plant. These datasets are searchable and can be downloaded conveniently via the PPMdb website. The statistical records of all analyzed plant genomes within the PPMdb, including the number of mapped pathways and designed primers, are summarized in Table 1.

**Table 1:** Summarize the number of mapped pathways and the number of designed primers of each plant genome.

Plant Name	No. of mapped pathways	No. of designed primers	Plant Name	No. of mapped pathways	No. of designed primers
*Arabidopsis lyrata*	121	27,138	*Lupinus angustifolius*	121	36,373
*Arabidopsis thaliana*	120	28,598	*Malus domestica*	121	34,730
*Arachis duranensis*	121	28,876	*Manihot esculent*	121	29,493
*Arachis ipaensis*	121	29,122	*Medicago truncatula*	120	31,198
*Asparagus officinalis*	120	23,804	*Momordica charantia*	120	19,537
*Beta vulgaris*	120	21,396	*Nelumbo nucifera*	120	24,488
*Brachypodium distachyon*	121	29,339	*Nicotiana attenuata*	121	27,056
*Brassica napus*	121	82,544	*Nicotiana sylvestris*	120	26,301
*Brassica oleracea*	121	41,415	*Nicotiana tabacum*	120	48,326
*Brassica rapa*	121	41,458	*Nicotiana tomentosiformis*	120	26,408
*Cajanus cajan*	121	29,112	*Olea europaea*	121	30,611
*Camelina sativa*	121	71,883	*Oryza brachyantha*	121	27,551
*Capsella rubella*	121	26,457	*Oryza sativa Japonica*	121	38,757
*Capsicum annuum*	121	26,524	*Papaver somniferum*	120	46,150
*Carica papaya*	120	17,993	*Phalaenopsis equestris*	119	20,473
*Chenopodium quinoa*	120	39,628	*Phaseolus vulgaris*	121	27,001
*Cicer arietinum*	121	25,279	*Populus euphratica*	121	33,292
*Citrus clementina*	121	22,067	*Populus trichocarpa*	121	76,911
*Citrus sinensis*	121	23,316	*Prunus avium*	121	24,449
*Cucumis melo*	120	50,235	*Prunus mume*	121	23,764
*cucumis sativus*	120	22,641	*Prunus persica*	121	23,604
*Cucurbita maxima*	120	31,349	*PyrusXbretschneideri*	121	33,747
*Cucurbita moschata*	120	31,857	*Quercus suber*	121	47,644
*Cucurbita pepo*	120	32,658	*Raphanus sativus*	121	47,881
*Daucus carota*	121	28,750	*Ricinus communis*	121	21,616
*Dendrobium catenatum*	120	22,022	*Rosa chinensis*	121	26,410
*Durio zibethinus*	121	37,592	*Sesamum indicum*	121	25,735
*Elaeis guineensis*	120	31,528	*Setaria italica*	121	32,295
*Eucalyptus grandis*	121	29,917	*Solanum lycopersicum*	120	26,382
*Eutrema salsugineum*	121	26,003	*Solanum pennellii*	121	26,602
*Glycine max*	121	46,715	*Solanum tuberosum*	120	27,323
*Glycine soja*	121	47,199	*Sorghum bicolor*	121	33,047
*Gossypium arboreum*	121	33,110	*Spinacia oleracea*	120	21,937
*Gossypium hirsutum*	121	53,945	*Tarenaya hassleriana*	121	30,052
*Gossypium raimondii*	121	33,246	*Theobroma cacao*	121	22,544
*Helianthus annuus*	121	37,275	*Vigna angularis*	121	27,066
*Hevea brasiliensis*	121	33,813	*Vigna radiata*	121	26,690
*Ipomoea nil*	121	30,569	*Vigna unguiculata*	121	28,687
*Jatropha curcas*	118	66,782	*Vitis vinifera*	121	25,183
*Juglans regia*	120	29,028	*Zea mays*	121	35,395
*Lactuca sativa*	121	29,366	*Ziziphus jujuba*	121	26,484

### Insights

Advances in plant omics promise to transform the molecular markers research area, in which the main challenge will not be the development of novel markers rather than the optimum selection and validation of a group of useful functional markers from the big collection of candidates^32^. For many decades, genetic diversity and molecular breeding studies have involved a few pre-specified candidate markers/genes. This knowledge-based approach was found to run a high risk of missing critical genes related to interest traits.

Genes or gene families involved in a biological pathway are often switched on or off together to reflect a particular biological function or elucidate specific phenotypes^1^. In plants, complex economic traits such as yield, resistance to a particular disease, production of secondary metabolites, etc.,… have thus driven the scientists' needs for new ‘systems’-based approaches that can illuminate the molecular mechanisms underlying specific trait(s) rather than the effect of distinct genes^3^. Based on this deep understanding, this study's scientific vision provides a platform for the development of pathways-based markers toward designing future studies that aim to disentangle the causal biological pathways and their phenotypic reflections.

As a part of this vision, microsatellites have been utilized as one of the most common sources of genetic markers, which have served as a keystone for massive genetic studies due to their robust and unique features. Microsatellites, which demonstrate their efficiency in broad applications, such as diversity studies, genome mapping, molecular breeding, and molecular phylogeny^33,34^, were successfully implemented in our developed PGM pipeline to develop the PMTM system as a novel class of pathway-targeted markers. In silico microsatellite mining on a plant genome scale is expected to advance our understanding and elucidate the functional impact of microsatellites within biological pathways and, consequently, in the context of systems biology^35^. This study also aimed to develop and map pathway-based genetic marker panels that support and boost molecular breeding programs, genetic diversity, and genetic characterization studies. From this point of view, the value of our developed marker panels will need to be assessed in the context of availability and mining of biological pathway information to generate knowledge that is more actionable rather than more complex.

### Conclusion

In summary, we present PPMdb as a comprehensive database for pathway-based markers in plant genomes. To our knowledge, PPMdb is the first portal providing unique pathway-targeted marker panels not presented in any previous database. PPMdb classifies developed marker panels based on their biological functions. This advantage allows researchers to deeply focus on the functional roles of utilized markers and may explain many phenotypic variations in the future. Our developed PPMdb is substantially different from similar plant genetic marker databases. The PPMdb will regularly be updated by integrating any newly released plant genomes.

Furthermore, the PPMdb graphical user interface and functionality will always be enhanced and continuously supported with new tools and technologies. Overall, we believe that the PPMdb hub will serve as a starting point or cornerstone for pathway-targeted marker research. In addition, we believe that PPMdb will catch great attention from a wide range of plant scientists in different disciplines, including genetic diversity, species characterization, population genetics, genome mapping, and targeted trait improvement.

## Data Availability

PPMdb is an online free access database initiative available at the following link: (http://PPMdb.easyomics.org).
